# β-Adrenergic Receptor Signaling in Prostate Cancer

**DOI:** 10.3389/fonc.2014.00375

**Published:** 2015-01-12

**Authors:** Peder Rustøen Braadland, Håkon Ramberg, Helene Hartvedt Grytli, Kristin Austlid Taskén

**Affiliations:** ^1^Department of Tumor Biology, Institute of Cancer Research, Division of Cancer Medicine, Transplantation and Surgery, Oslo University Hospital, Oslo, Norway; ^2^Institute of Clinical Medicine, University of Oslo, Oslo, Norway

**Keywords:** ADRB2, β-adrenergic receptor, prostate cancer, neuroendocrine differentiation, angiogenesis, apoptosis, metastasis, β-blocker

## Abstract

Enhanced sympathetic signaling, often associated with obesity and chronic stress, is increasingly acknowledged as a contributor to cancer aggressiveness. In prostate cancer, intact sympathetic nerves are critical for tumor formation, and sympathectomy induces apoptosis and blocks tumor growth. Perineural invasion, involving enrichment of intra-prostatic nerves, is frequently observed in prostate cancer and is associated with poor prognosis. β_2_-adrenergic receptor (ADRB2), the most abundant receptor for sympathetic signals in prostate luminal cells, has been shown to regulate trans-differentiation of cancer cells to neuroendocrine-like cells and to affect apoptosis, angiogenesis, epithelial–mesenchymal transition, migration, and metastasis. Epidemiologic studies have shown that use of β-blockers, inhibiting β-adrenergic receptor activity, is associated with reduced prostate cancer-specific mortality. In this review, we aim to present an overview on how β-adrenergic receptor and its downstream signaling cascade influence the development of aggressive prostate cancer, primarily through regulating neuroendocrine differentiation.

## Introduction

Most men die with and not from prostate cancer. Despite this, prostate cancer was the primary cause of death in more than 300,000 men worldwide in 2012, with an estimated 630,000 deaths to be expected in 2035 (1). Neuroendocrine prostate cancer, a poorly defined clinical phenotype of aggressive disease, is predicted to cause approximately 10–25% of the prostate cancer-specific deaths (2–4). Drugs targeting androgen receptor activity promote development of a neuroendocrine prostate cancer phenotype (5) and increases the prevalence of neuroendocrine cells (6), and as more drugs in this category reach the clinic the occurrence is expected to rise. Neuroendocrine-like cancer cells are differentiated to a varying extent and may express luminal, mesenchymal, and/or stem cell markers in addition to neuroendocrine markers (7–11). This reflects the high plasticity of these cells. Although the molecular mechanisms underlying neuroendocrine differentiation *in vivo* are poorly understood, inflammation (12), androgen deprivation (13), ionizing radiation therapy (14), and activation of the β-adrenergic receptor (ADRB) have been shown to induce trans-differentiation of prostate cancer cell lines to neuroendocrine-like cells *in vitro*.

Over the last decade, epidemiologic studies have indicated that use of β-blockers may have beneficial effects on cancer progression, metastasis, and mortality (15–24). β-blockers form a group of commonly prescribed drugs used as treatment for hypertension, cardiac heart failure, and arrhythmias, as well as for migraine prophylaxis. In two Norwegian cohorts of patients with aggressive prostate cancer, it was reported that use of β-blocker was associated with reduced prostate cancer-specific mortality (21, 22). In contrast, a nested case-control study of prostate cancer patients in the UK Clinical Practice Research Datalink cohort did not observe an effect of β-blocker usage after diagnosis on prostate cancer-specific deaths (25). However, use of β-blocker has been reported to be inversely associated with progression of breast, ovarian, and non-small cell lung cancer (23). Moreover, β-blocker use has been associated with longer relapse-free survival (15) and lower risk of tumor recurrence (17), distant metastasis (17), and cancer-specific mortality (16, 17) in breast cancer patients. Indeed, pre-clinical and epidemiological evidence have led to the initiation of clinical phase II studies evaluating the effect of administering the β-blocker propranolol to ovarian, cervix, colorectal, and breast cancer patients (ClinicalTrials.gov identifiers: NCT01504126, NCT01308944, NCT01902966, NCT00888797, and NCT01847001). Together this indicates that more studies on prostate cancer cohorts are needed.

In this review, we will focus on how β-adrenergic activity, primarily via the β_2_-adrenergic receptor (ADRB2) and the subsequent cyclic AMP (cAMP) signaling pathway, affects development of aggressive prostate cancer by regulating neuroendocrine differentiation, metastasis, angiogenesis, and apoptosis-resistance.

## Adrenergic Receptor’s Functional Role in the Prostate

The β-adrenergic receptors (ADRBs) are part of the sympathetic nervous system, the general role of which is to ensure that the body responds fast and targeted upon danger, as well as to regulate the whole body energy expenditure. The receptors are activated by catecholamines; norepinephrine released by adrenergic nerves, innervating most major organs, and epinephrine produced by chromaffin cells (26). Chromaffin cells are most highly abundant in the adrenal medulla, but paraganglia has also been observed in proximity to sympathetic nerves within the prostate (27, 28). Macrophages, aside from exerting an immunosuppressive activity following catecholamine stimulation (29), also have the capacity to produce catecholamines themselves to a minor extent (30). Interestingly, infiltration of macrophages has been reported to be associated with prostate cancer aggressiveness (31, 32).

The prostate is highly innervated (33), and the nerves are required for formation of the prostate during embryogenesis, maturation during puberty, and maintenance of the adult phenotype (34). Thus, like androgen stimulation, sympathetic stimuli contribute to prostatic differentiation *in vivo* (35). Interestingly, most prostate cancers originate from the peripheral zone, which is part of the posterior region where the majority of nerves are located (36). Whereas, parasympathetic nerves are uniformly spread from the base to the apex and innervate the epithelium, sympathetic nerves are slightly enriched toward the base and are in close contact with the smooth muscle cells (36–39). The adrenergic nerves fire during ejaculation, promoting contraction of smooth muscle cells expressing α-adrenergic receptors (40). In addition, adrenergic stimulation facilitates secretion from the luminal cells predominately expressing β-adrenergic receptors (41).

The interplay between nerves and cancer cells is an emerging field in prostate cancer research. Intact sympathetic nerves were recently shown to be essential for tumor formation as sympathectomy induced apoptosis and blocked prostatic intraepithelial neoplasia formation and tumor growth in a mouse model (42). Furthermore, perineural invasion is a phenomenon whereby cancer cells are frequently observed to surround or track the nerve fiber (43). An increasing number of studies conclude that perineural invasion is a prognostic marker in prostate cancer (44, 45). The nerve density is enriched in cancer areas and higher in prostatic tissue from high-risk compared to low-risk prostate cancer patients (42, 46), indicating that neurogenesis may occur during cancer development (47).

Prostate cancer cells proximal to areas of perineural invasion have been shown to exhibit reduced apoptosis and increased proliferation compared to distant cancer cells (47). In a prostate cancer case study, increased frequency of neuroendocrine-like cells was observed in the proximity to perineural invasion (48). Interestingly, in a pancreatic cancer study, catecholamine exposure from co-cultured dorsal root ganglia was shown to promote perineural invasion both *in vitro* and in animal experiments (49). A possible mechanism explaining this observation is that norepinephrine secreted by the sympathetic nerves acts as a chemoattractant, promoting cancer cell migration toward innervated areas (49), with subsequent metastasis through the perineural space. Studies are wanted to unravel whether this mechanism is involved in stress-induced metastatic prostate cancer.

## ADRB2 Regulation and Downstream Signaling in Prostate Cancer

The prostate is highly enriched in β-adrenergic receptors with ADRB2 being the dominating isoform in luminal cells [ADRB1: (50, 51); ADRB2: (41, 51–53); and ADRB3: (54)]. More than 95% of the β-adrenergic receptor binding activity in PC-3 cells is mediated through ADRB2 (51), and the main ADRB isoform in LNCaP cells is the β_2_ subtype (52). β_2_- and β_3_-adrenergic receptors have been observed in stromal cells (55, 56), although immunohistochemical staining using ADRB2 antibodies showed predominantly epithelial localization in both benign and malignant prostate tissue (57, 58). In the first immunohistochemical staining report of β_2_-adrenergic receptor in human prostate, ADRB2 was only observed in malignant tissue (59). Most gene expression profiles show up-regulation of ADRB2 mRNA in prostatic adenocarcinomas (57, 60), and the general consensus in the literature is that the protein expression level of ADRB2 is increased in prostate cancer cells compared to benign prostate cells (57, 58). Following castration in mice and during androgen deprivation therapy of prostate cancer patients, low β-adrenergic activity, and down-regulation of ADRB2 mRNA, respectively, has been reported (57, 61). Although ADRB2 is up-regulated in malignant cells, the expression level seems to decrease during progression as ADRB2 is inversely correlated with PSA recurrence-free survival (58). In metastatic prostate cancer, the situation is more complex as both high and low levels of ADRB2 have been observed (57, 58). ADRB2 is assumed to be up-regulated in castration-resistant prostate cancer to support sensitization of the androgen receptor, but it is down-regulated in the androgen independent sub-line LNCaP-abl at the mRNA-level (62), and at the protein level in LNCaP-Rf (57), both compared to the parental LNCaP cell line. Amplification of ADRB2 has, however, been reported in 3 out of 28 cases in a cohort of castrated metastatic prostate cancer patients (60). More data are needed to test whether ADRB2 is involved in development of castration-resistant prostate cancer.

Besides being regulated by thyroid hormones in LNCaP cells (57), *ADRB2* has been shown to be an androgen receptor target gene (35, 63–65). Interestingly, *ADRB2* is also a target gene of two important markers in prostate cancer that are involved in transcriptional regulation; v-ets avian erythroblastosis virus E26 oncogene homolog (ERG) (66) and Enhancer of zeste homolog 2 (EZH2) (58). Both ERG and EZH2 exert repressive action on *ADRB2* transcription *in vitro*, through direct binding and epigenetic silencing, respectively (66). Furthermore, ERG up-regulates the expression of *EZH2* (66). This suggests that ERG and EZH2 antagonize the stimulatory effect of androgen on ADRB2 expression. The overall effect, however, based on analysis of data from cBioPortal is that ADRB2 as well as ERG and EZH2 are either up-regulated or unaltered at the mRNA level in malignant compared to benign prostate tissue (60). This does not rule out the possibility that ERG and/or EZH2 exert a more dominating effect on ADRB2 expression, as suggested by Yu et al. (58, 66), in advanced diseases. ERG was recently shown to inhibit luminal and neuroendocrine differentiation in a transgenic prostate cancer mouse model (67), suggesting that ERG can be linked to de-differentiation of cancer cells. This would fit into the hypothesis that ADRB2 is positively and ERG negatively correlated with a differentiated phenotype. Although the prognostic value of TMPRSS2-ERG is controversial, this hypothesis would also support a role of ERG as prognostic marker (67–69).

**Figure 1:**
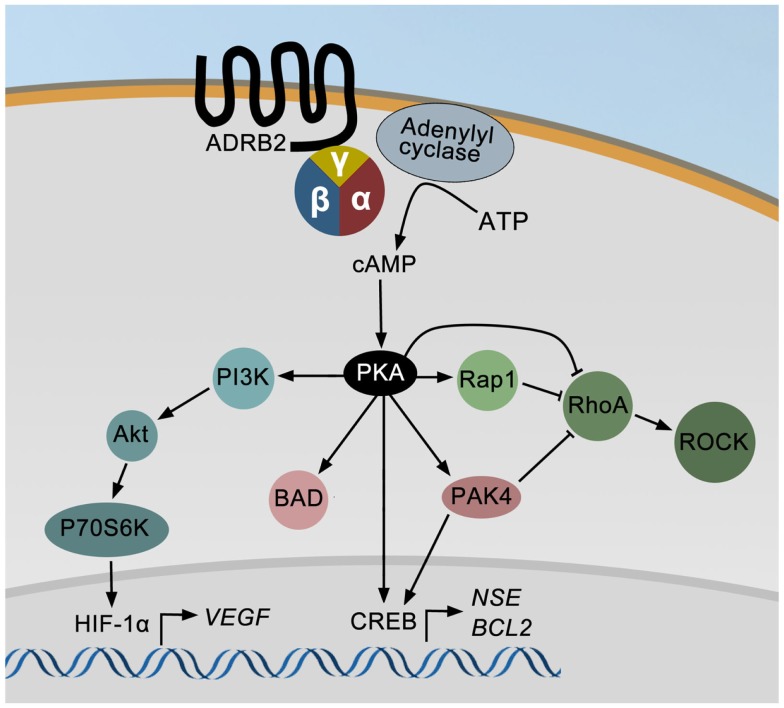
**The ADRB2 signaling pathways in prostate cancer**. Ligand binding to ADRB2 increases the intracellular level of cAMP, which activates cAMP-dependent protein kinase (PKA). PKA may either directly or through PAK4 stimulate CREB activity and thereby induce the expression of *ENO2* and *BCL2*. PKA can also directly or indirectly via PAK4 or Rap1 inhibit RhoA and ROCK activities and thereby induce neurite outgrowth. Finally, *VEGF* expression is up-regulated by adrenergic stimulation via PI3K/AKT/p70S6K mediated activation of HIF-1α.

An overview of known ADRB2 downstream signaling pathways in prostate cancer cell lines is summarized in Figure 1. ADRB2 is a seven-trans membrane G-protein coupled receptor primarily acting through the cAMP-signaling pathway. Ligand binding to ADRB2 stimulates adenylyl cyclase activity and cAMP production via Gα_s_. Induction of cAMP in response to adrenergic stimulation has been shown in a number of prostate cancer cell lines (70–73). Most effects of cAMP are mediated through the cAMP-dependent protein kinase (PKA), and among other proteins regulated by cAMP are exchange proteins activated by cAMP (EPAC) and cyclic nucleotide-gated ion channels. It is not known whether these are activated in response to adrenergic stimulation of prostate cancer cells, but activation of EPAC, using an EPAC-specific cAMP analog, affects the MAP kinase, RhoA (74), and AKT-p70S6K signaling pathways in prostatic epithelial cells (75, 76). These pathways are also regulated by adrenergic activation in prostate epithelial cells as described below. Noteworthy, treatment of LNCaP cells with an EPAC analog indicated that PKA is the dominating mediator of neuroendocrine differentiation in these cells (77).

Cyclic AMP produced in response to adrenergic stimulation binds the regulatory subunit of PKA and the activated catalytic subunit is released. The catalytic subunit may translocate to the nucleus and phosphorylate cAMP responsive element binding protein (CREB), which induces the expression of e.g., neuron specific enolase/enolase 2 (*ENO2*, a neuroendocrine marker), and B-cell CLL/lymphoma 2 (*BCL2*, encoding an anti-apoptotic protein) (78). PKA-induced phosphorylation of CREB may either be direct or indirect through regulation of p21-activated protein kinase 4 (PAK4) and/or ERK activity. Stress may also promote apoptosis-resistance through PKA-dependent phosphorylation of BCL2-associated agonist of cell death (BAD), as shown in Figure 1 (79). Furthermore, PKA may inhibit the ras homolog family member A (RhoA) – Rho-associated PKA (ROCK) pathway leading to neurite outgrowth either directly or mediated through either Rap1, a member of the RAS oncogene family, or PAK4 (80). Rap1 is also possibly involved in PKA-induced regulation of ERK activity (not shown in Figure 1). Finally, PKA-mediated effects of adrenergic stimuli up-regulate vascular endothelial growth factor (VEGF) levels and HUVEC capillary tube formation via the PI3K/AKT/p70S6K/HIF-1α pathway (81).

Besides regulating the transcription factor activity of CREB and HIF-1α, the ADRB2/cAMP/PKA signaling pathway has been shown to stimulate the androgen receptor responsive gene transcription (57, 72). The putative molecular mechanisms involved in ADRB2/PKA-mediated regulation of androgen receptor activity have been thoroughly described in a review by Merkle and Hoffmann (82).

Much is still to be learned about the ADRB2 signaling pathway in prostatic luminal cells. β-arrestin is instrumental in the desensitization and internalization/sequestration of β-adrenergic receptors (83). One study reported increased formation of a β-arrestin-SRC complex following ADRB2 stimulation in LNCaP cells over-expressing β-arrestin2 (73). How this affects the functional effects of adrenergic signaling is unknown.

## Adrenergic Regulation of Neuroendocrine Differentiation

β-adrenergic stimulation is a well-known inducer of neuroendocrine differentiation of prostatic adenocarcinoma cell lines (70, 84–86). Data linking sympathetic stimuli to neuroendocrine differentiation *in vivo*, however, are currently lacking. Cox and co-workers reported in a series of publications that the ADRB2 agonists epinephrine and isoproterenol caused a rise in the intracellular cAMP levels, followed by increased activity of cAMP-dependent PKA and a higher number of neuroendocrine-like cancer cells (70, 84). cAMP has been shown to induce neuroendocrine differentiation to various extent in multiple prostate cancer cell lines; namely LNCaP, PC-3, and PC-3-M (70, 85, 87–89). Furthermore, neuroendocrine differentiation of LNCaP cells was observed when the cells were transfected with a plasmid expressing a constitutive active PKA catalytic subunit (84). The induction of a neuroendocrine-like morphology was inhibited after transfection of the LNCaP cells with a PKA regulatory subunit containing mutations that rendered the PKA holoenzyme complex in an inactive state despite increased cAMP levels. Moreover, cAMP-signaling has been reported to up-regulate neuropeptides like PTHrP and neurotensin in LNCaP cells (70).

Interestingly, the first evidence of different substrate specificity between the various isoforms of the catalytic subunit of PKA was observed in prostate cancer cells (90). Prostate cancer cells express both the ubiquitously expressed C_α_ subunit and the cell-type specific C_β_ isoforms (C_β1_, C_β2_, C_β3_, and C_β4_) of PKA (91). The PKA C_β2_ subunit has previously been shown to be up-regulated in the more proliferating prostate epithelial cells present in malignant compared to benign prostate tissue (90). Up-regulation of MYC is an early event during prostate tumorigenesis and PKA C_β2_ has been shown to be a MYC target gene and to participate in a positive feedback loop whereby MYC is stabilized (90). Prolonged activation of PKA C_α_, however, represses MYC transcription and may thereby promote growth arrest and neuroendocrine differentiation. In contrast, the PKA C_β2_ splice variant has only a minor effect on MYC transcription and is supposed to be linked to the growth stimulatory effect of MYC. Although it is unknown whether any specific PKA isoforms act downstream of ADRB2, the overall effect of adrenergic stimulation of LNCaP cells is inhibition of proliferation; indicating that C_α_ is mediating the effect (70, 92). Similarly, growth arrest was observed after cAMP treatment in PC-3-M cells, suggesting that C_α_ plays a dominating role (85). Generally, the anti-mitogenic effect of ADRB stimulation involving cAMP is in agreement with the non-mitotic characteristic of most neuroendocrine cells.

Neurite outgrowth, a dynamic process in which actin rearrangements cause the cells to obtain a more neuronal phenotype, can be observed in LNCaP cells after 3–5 days of incubation in charcoal-stripped serum (mimicking androgen deprivation) (93). These morphological changes occur simultaneously with a rise in cAMP (6), linking neurite outgrowth to the before-mentioned cAMP-induced neuroendocrine differentiation. Upon adrenergic stimulation, neurite outgrowth is observed as early as after 1 hour (70). Activation of ADRB has been shown to induce an immediate increase in cAMP, which could explain the more rapid appearance of a neuronal phenotype (70) as compared to the delayed increase following androgen-depletion.

Cytoskeletal rearrangements are essential in the process of neurite outgrowth, and are regulated by small Rho GTPases like CDC42, Rac1, and RhoA, each controlling distinct morphogenic pathways. Inactivation of RhoA promotes neurite outgrowth in neuronal cells (94, 95). One possible mechanism by which ADRB/cAMP/PKA regulate these cytoskeletal rearrangements involved in neurite outgrowth is through direct inactivation of RhoA (80, 94), as illustrated in Figure 1. In LNCaP cells, the RhoA inhibitor C3 transferase was reported to induce trans-differentiation to neuroendocrine-like cells (77, 96). Furthermore, inhibition of the RhoA downstream effector ROCK has been shown to induce neurite outgrowth in PC-3 cells and to a lesser extent also in LNCaP cells (97). A similar effect is seen through PAK4-induced activation of RhoA as shown in Figure 1 (80). PAK4 may also mediate the effect of ADRB2/cAMP/PKA on neuroendocrine differentiation in prostate cancer cells by regulating the activity of the transcription factor CREB (78). PKA has been shown to activate PAK4 through phosphorylation, which induced the transcriptional activity of CREB and thereby the expression of NSE/ENO2.

In general, assembly of stress fibers plays an important role in adhesion and motility of eukaryotic cells and loss of stress fibers is associated with neurite outgrowth and reduced migratory capacity (98). Upon destabilization of stress fibers, the cell experiences cytoskeletal alterations and loss of focal adhesions, both required for the cell to migrate. Maintenance of stress fiber integrity is ensured through inhibition of actin filament depolymerization and is regulated by the RhoA/ROCK pathway (99). In addition, PKA has been shown to phosphorylate actin monomers directly, thereby destabilizing the stress fibers (100). These mechanisms have not been explored in prostate cancer models.

## ADRB2 Expression and Effects on Metastasis

Most prostate cancer metastases are detected in bone, lymph nodes, lung, and liver (101). Metastasis is a complex multi-step process involving the ability of cancer cells to detach from the primary tumor site, degrade extracellular matrix, migrate to other parts of the body, and to invade and settle at the metastatic site (102). The requirement for different properties is constantly changing during the metastatic process, favoring cells with high plasticity. Whereas, de-differentiation like epithelial–mesenchymal transition (EMT) promotes detachment and migration, re-differentiation, or mesenchymal–epithelial transition favors homing to metastatic sites.

In the work by Yu and colleagues, it was shown that the expression level of ADRB2 changes during the metastatic process in prostate cancer (58). Although up-regulation of ADRB2 is observed in malignant compared to benign prostate tissue (57), a decrease in ADRB2 expression is observed in aggressive relative to indolent prostate cancer (58). Interestingly, knockdown of ADRB2 was shown to induce EMT of transformed prostatic epithelial cells (RWPE-1). Expressional analyses revealed that the ADRB2 knockdown cells acquired an increased expression of vimentin (*VIM*) and N-cadherin (*CDH2*), as well as lowered expression of β-catenin (*CTNNB1*) and integrin β4 (*ITGB4*) suggesting that the cells harbor a mesenchymal-like phenotype. The ADRB2 knockdown cells, as well as cells treated with an ADRB2 antagonist (ICI 118,551), showed increased ability to migrate and invade. Conversely, treatment with an ADRB agonist, isoproterenol, reduced invasion in these cells as well as in DU145 cells (58).

In a PC-3 xenograft mouse model, however, norepinephrine promoted metastasis (59). This might be due to increased migration, as suggested in a study by Lang et al. where increased migratory activity in PC-3 cells was observed upon norepinephrine stimulation (103). The effect was partially inhibited by treating the cells with the β_1_-specific β-blocker atenolol, and fully inhibited with the β_2_-specific blocker ICI 118,551. Furthermore, in a xenograft model using ADRB2 and ADRB3 double knockout mice (*ADRB2*^−/−^, *ADRB3*^−/−^), lowered human tumor cell dissemination to lymph nodes and distant organs was observed (42). Whether stromal ADRB2 and ADRB3 affect the metastatic process could not be addressed in this model system as tumor development was severely compromised in *ADRB2*^−/−^, *ADRB3*^−/−^ mice.

The ADRB2 expression level affects the phenotype of the prostate cells and thereby their ability to migrate and invade (58), and probably also their ability to settle at the metastatic site, which would indicate a role of ADRB2 in the whole metastatic process. Low expression of ADRB2 in prostatic epithelial cells is associated with a mesenchymal-like phenotype (58). These cells may have the potential to re-differentiate into epithelial cells adapted to the microenvironment at the metastatic site. To what extent this involves up-regulation of ADRB2 and development of neuroendocrine-like tumors at the metastatic site is currently not known. Interestingly, adrenergic stimulation has been linked to pro-angiogenic processes in different cancer models (81, 104), and may thus aid in providing the cancer cells with another mean to escape the primary tumor site.

## Stress-Induced Regulation of Angiogenesis

Neuroendocrine cells are the primary site of VEGF production within the prostate (105). It is therefore compelling that the number of neuroendocrine cells present in high-grade prostatic carcinoma correlates with the degree of neovascularization (106, 107). Studies have shown that neuroendocrine cells promote growth of neighboring cancer cells through secretion of neuropeptides (108–111), and consequently an increased energy supply through the blood stream is required by the tumor. The fact that several factors are involved in both angiogenesis and neuroendocrine differentiation is intriguing, and points at a possible linkage between the two processes.

Chronic stress has been reported to increase tissue norepinephrine levels in an ovarian carcinoma mouse model, resulting in the formation of new blood vessels, and increased expression of the pro-angiogenic VEGF (104). Similar observations have been reported in the androgen-sensitive LNCaP cell line, where VEGF-expression increased in a dose-dependent manner upon ADRB2-mediated epinephrine stimulation (112). Furthermore, in androgen-insensitive PC-3 cells, norepinephrine, and isoproterenol stimulation induced VEGF expression through increased activity of the cAMP/PKA pathway (81). Interleukin 6 (*IL6*), a well-known inducer of neuroendocrine differentiation, and a putative downstream target of adrenergic signaling, also functions as a pro-angiogenic factor (92, 113, 114). Moreover, conditioned media from norepinephrine stimulated PC-3 cells induced HUVEC capillary tube formation in an *in vitro* angiogenesis assay (81). In concordance with the adrenergic stimulatory effects on VEGF and capillary tube formation, treatment of rats with propranolol resulted in a reduction in ventral prostate blood vessel volume (115). Hassan and colleagues were, however, not able to detect a significant up-regulation of plasma-VEGF levels in stressed compared to calm mice (116). They also measured the micro-vessel density in Hi-Myc mice after stress-induced adrenergic stimulation, and also here they did not observe any significant difference between calm and stressed Hi-Myc mice.

Although there are very few studies that have addressed the effects of ADRB stimulation on angiogenesis in prostate cancer, the strong evidence from other model systems (117–119) warrants further investigation into this field in different prostate cancer models.

## Adrenergic Regulation of Apoptosis

Stress has been reported to reduce apoptotic activity (116, 120), whereas sympathectomy increases apoptosis in mouse prostate cancer models (42). Thus, prolonged elevation of catecholamines may promote prostate cancer progression by inducing resistance to apoptosis. This is in agreement with the observation that epinephrine protects LNCaP and C4-2 cells from apoptosis induced by the PI3K inhibitor LY294002, and thapsigargin (79).

Adrenergic signaling regulates apoptotic activity by multiple mechanisms as indicated in Figure 1. The best characterized mechanism in prostate cancer cells is the PKA-mediated phosphorylation of BAD on Ser112 and Ser155 (79, 121, 122). Increased phosphorylation of BAD at Ser112 was also observed in mice, and may explain the stress-induced resistance to apoptosis observed in mouse models (116). PAK4 has also been shown to phosphorylate BAD on Ser112 in HeLa cells over-expressing PAK4 (123). Furthermore, PKA induces apoptosis-resistance by directly or indirectly activating CREB and thereby up-regulating the level of BCL2 (78). The anti-apoptotic BCL2 protein acts downstream of ADRB2 in pancreatic cancer cells (124).

Repeated immobilization stress, which elevates the plasma level of epinephrine and inhibits apoptosis, has been shown to accelerate cancer development in mice through stimulation of β-adrenergic receptors (116). Prolonged elevation of catecholamines is also observed in obese (125, 126) and chronically stressed (127, 128) individuals, and may represent one mechanism by which obesity, and perhaps stress, promote development of aggressive prostate cancer (129–131). As a negative feedback mechanism, enhanced levels of catecholamines may lead to down-regulation of ADRB2. Interestingly, expression of ADRB2 in prostatectomy specimens is inversely correlated with biochemical recurrence (BCR) (58), suggesting that chronic stress and low levels of ADRB2 are associated with disease progression. Studies are needed to enlighten this hypothesis.

## Controversies, Clinical Implications, and Conclusions

**Figure 2:**
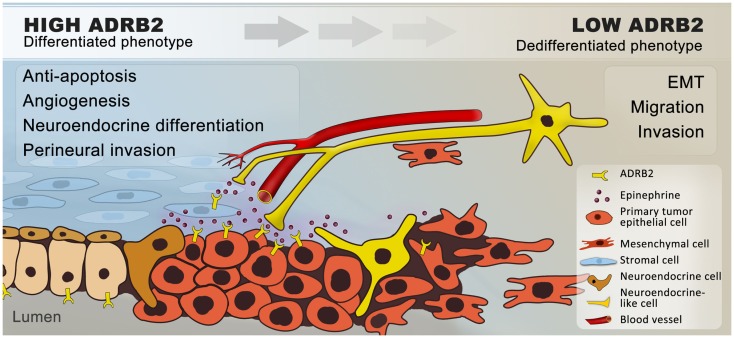
**Hypothetical model of how β_2_-adrenergic signaling may promote progression of prostate cancer**. In summary, we hypothesize that ADRB2s expressed on luminal cells are activated by catecholamines, which are secreted by nerves and transported through blood vessels in response to stress. Catecholamines are possibly also secreted by proximal chromaffin-like cells and macrophages (not shown) that also can produce epinephrine and norepinephrine, respectively. In addition, ADRB2s expressed on stromal cells are activated by sympathetic stimuli. Upon ligand-binding, the expression of anti-apoptotic and pro-angiogenic factors is increased and a number of cancer cells undergo trans-differentiation to neuroendocrine-like cells. Together this will favor tumor growth. Angiogenesis and neurogenesis are closely linked (132) and sympathetic activation may stimulate perineural invasion through chemotaxis. In general, chronic ADRB2 activation down-regulates the ADRB2-level, leading to de-differentiation and epithelial–mesenchymal transition, with a subsequent increase in the migratory and invasive potential of the cells. Cancer cells expressing low levels of ADRB2 will thereby follow the nerves and blood vessels to metastatic sites.

In normal prostate physiology, the sympathetic nervous system regulates prostate differentiation and secretory activity of luminal cells, predominantly through ADRB2 (34, 35, 40, 41). We know from *in vitro* and *in vivo* prostate cancer models that chronic elevation of ADRB activity by exposing mice to repeated stress or by adding ADRB agonists promotes neuroendocrine differentiation (70, 84–86), metastasis (58, 103), angiogenesis (78, 81, 112, 115), and apoptosis-resistance (116, 120); together indicating that adrenergic signaling promotes prostate cancer progression (Figure 2).

**Figure 3:**
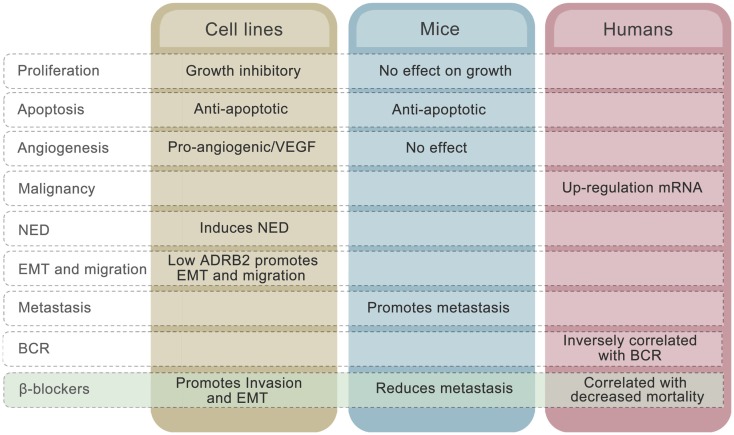
**Effects of ADRB2 on tumor characteristics in cell lines, mouse models, and human prostate cancer**. The effects of β-blockers on the different characteristics in each model system is also shown.

There are, however, several controversies in the field that challenge this hypothetical model. To begin with, the collective evidence fails to point in any obvious direction in terms of whether adrenergic signaling is beneficial or disadvantageous for prostate cancer patients. Both stimulatory and inhibitory effects of adrenergic stimulation on proliferation have been observed in cell line studies (70, 73, 85, 92). The majority of publications involving prostate cancer cell lines, however, claim that elevated β-adrenergic receptor activity induces growth arrest *in vitro* and has no effect in mouse models, alongside undergoing neuroendocrine differentiation in cell lines (116). Furthermore, adrenergic signaling up-regulates VEGF expression (112) and promotes HUVEC capillary tube formation in cell line experiments (81). Induction of anti-apoptotic mechanisms through ADRB2 stimulation has been seen in both cell lines and in prostate cancer xenograft models (116). To what extent these mechanisms are involved in development of human prostate cancer is unknown, but reduced apoptotic activity and stimulation of angiogenesis may be consequences of up-regulated ADRB2 levels, which are seen in malignant compared to benign prostatic epithelial cells (Figure 3).

The mechanisms described above cannot explain why low rather than high level of ADRB2 is associated with disease progression measured as BCR. The inverse correlation between ADRB2 and BCR may relate to the observation that, whereas high ADRB2 activity induces neuroendocrine differentiation, low ADRB2 activity/level promotes EMT in prostate cell lines (Figure 3). Cancer cells expressing low levels of ADRB2 have a mesenchymal-like phenotype and have a higher probability of being in the circulation at time of prostate removal (radical prostatectomy). These cells may also have a higher degree of plasticity and will therefore more easily adapt to environmental changes and thereby produce recurrent tumors in both humans and mice. The fact that reduction in adrenergic activity induces EMT in prostatic epithelial cells gives rise to another conflicting observation, since adrenergic stimulation promoted metastasis in a PC-3 xenograft model (59). A plausible explanation to this is that circulating tumor cells are already present in the xenograft model due to the mesenchymal-like phenotype of PC-3 cells and that binding of ligand to ADRB promotes mesenchymal to epithelial transition (MET) and homing to metastatic sites. Along the same line, we may explain the β-blocker paradox. In cell line experiments, β-blockers have been shown to promote EMT (58) whereas use of β-blocker is associated with reduced mortality in prostate cancer patients (21, 22). Again, inhibition of MET by β-blockers is one hypothesis that needs to be unraveled.

The reports on effects of β-blockers on mortality in other cancer types brings forth an important question: are the *in vivo* effects of β-blockers mediated by common tissue specific/non-specific attributes, or are the effects indirect (i.e., systemic or neural effects facilitated by other local or distant tissue expressing ADRBs)? β-blockers probably have an effect on immune responses, hormone levels, angiogenesis, neurogenesis, and at the metastatic niche. In the prostate, stromal cells proximal to tumor tissue express ADRBs, and may exert the effect, which may also explain the discrepancy between cell line results and *in vivo* data. It is also worth noting that the majority of β-blockers are targeting β_1_-adrenergic receptors or both β_1_- and β_2_-adrenergic receptors, whereas ADRB2 has been the receptor mediating the effects on cancer cells. Another plausible explanation lies in the antagonistic mechanism of action. Propranolol, for example, a commonly used antagonist *in vitro*, has been shown to function as an inverse agonist (133), and can thus lower the β-adrenergic receptor’s activity below its’ basal level. In clinical practice, however, numerous β-blockers are used, and their mechanisms of action vary. Furthermore, the differences observed could be dose-dependent, as it is difficult to measure the dose in patient tissue, whereas this parameter can be controlled in cell lines and animal models. We anticipate that ADRB antagonists will reduce the development of neuroendocrine prostate cancers, but this has not yet been addressed in any publications. More studies are needed to unravel whether β-blockers can play a role in future tailored prostate cancer therapy.

ADRB2 may play a role both as a prognostic and as a predictive biomarker in prostate cancer. We do not know, however, whether the expression level of ADRB2 is a driver of progression. Still, it is plausible to hypothesize that the receptor may be involved in maintenance of a differentiated phenotype, an attribute that is lost when the cells gain plasticity and metastasize, and the disease reaches an incurable stage. We know that ADRB2 is inversely correlated with time to BCR and that it acts independently of Gleason score, surgical margin status and preoperative PSA as a prognostic marker (58). Actually, ADRB2 was the strongest predictor of clinical failure in the study by Yu et al. Validation studies also addressing a potential association with metastasis, development of castration resistance, and survival is warranted to determine whether ADRB2 is a clinically relevant prognostic marker in prostate cancer. The fact that β-adrenergic signaling induces neuroendocrine differentiation and apoptosis-resistance of prostate cancer cells suggest that ADRB2 could play a role in predicting responsiveness to pro-apoptotic drugs.

## Author Contributions

All authors (Peder Rustøen Braadland, Håkon Ramberg, Helene Hartvedt Grytli, and Kristin Austlid Taskén) have contributed to the design, drafting, and approval of the manuscript.

## Conflict of Interest Statement

The authors declare that the research was conducted in the absence of any commercial or financial relationships that could be construed as a potential conflict of interest.
